# Microbial Decontamination of Bee Pollen by Direct Ozone Exposure

**DOI:** 10.3390/foods10112593

**Published:** 2021-10-27

**Authors:** Juan Ramón Cabello, Salud Serrano, Inmaculada Rodríguez, Ana Isabel García-Valcárcel, María Dolores Hernando, José Manuel Flores

**Affiliations:** 1 Department of Food Science and Technology, University of Córdoba, 14071 Córdoba, Spain; juanramon.cabellocivico@gmail.com (J.R.C.); v62rodem@uco.es (I.R.); 2 National Institute for Agricultural and Food Research and Technology (INIA), 28040 Madrid, Spain; aigarcia@inia.es (A.I.G.-V.); hernando.dolores@inia.es (M.D.H.); 3 Department of Zoology, University of Córdoba, 14071 Córdoba, Spain; ba1flsej@uco.es

**Keywords:** bee pollen, ozone, microbial decontamination, polyphenols, sensory analysis

## Abstract

The bee pollen is a complete and healthy food with important nutritional properties. Usually, bee pollen is consumed dehydrated, but it is possible to market it as fresh frozen pollen, favoring the maintenance of its properties and greatly increasing its palatability, compared to dried pollen. However, fresh frozen pollen maintains a high microbiological load that can include some pathogenic genus to human health. In this work, ozonation combined with drying is applied to reduce the microbiological load. The lowest timing exposure to ozone (30 min) was chosen together with hot-air drying during 15 min to evaluate the shelf-life of treated bee-pollen under cold storage (4 °C), and initial reductions of 3, 1.5, and 1.7 log cycles were obtained for *Enterobacteriaceae*, mesophilic aerobes, and molds and yeasts counting, respectively. Six weeks after treatment the microbial load was held at a lower level than initially observed in fresh bee-pollen. In addition, ozone treatment did not have a negative impact on the polyphenols evaluated. Likewise, the sensory profile of the bee pollen under different treatments was studied. For all these assays the results have been favorable, so we can say that ozonation of fresh pollen is safe for human consumption, which maintains its polyphenols composition and organoleptically is better valued than dried pollen.

## 1. Introduction

The honey bee (*Apis mellifera* L.) is a social insect that belongs to order Hymenoptera. In every honey bee colony, there are three castes: queen, workers, and drones. Honey bees’ colonies are permanent, survive the winter and, in favorable environment, they are reported to live up to several years. While floral nectar and other plant secretions provide the colony with the main source of sugars, pollen supplies proteins, fats, and other nutrients. As permanent colonies, honey bees need to stock up on food to cope with adverse conditions. Because of this, they collect plenty of nectar and pollen, which later on are stored in empty comb cells, as honey and beebread, respectively.

**Figure 1:**
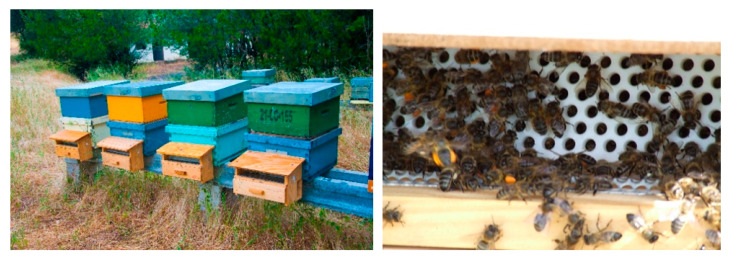
Pollen-traps.

Indeed, that is the basis of honey and pollen production in beekeeping. Honey bees work hard and often produce more honey than they need, which allows beekeepers to harvest the excess. The production of bee-pollen is carried out when beekeepers assemble pollen-traps at the entrance of the beehive to collect the pollen pellets that honey bees carry in the pollen basket of their hind legs (also known as corbicular pollen). When foraging, honey bees become covered with pollen. To groom themselves, they use stiff structures on their legs and brush the pollen off their bodies. The pollen grains are mixed with some regurgitated nectar and bee salivary secretions before packing all of them together into small pellets to be transported on their rear legs. By returning, bees enter the hive through the small holes of pollen traps and the pellets of pollen are scraped off from the legs and fall into a collecting drawer beneath (See Figure 1) [1,2,3,4,5].

The bee-pollen has beneficial properties to human health. It is considered a highly nutritious food because of its balanced content of proteins, free amino acids, vitamins, minerals among others. The corbicular pollen is a pack of pollen grains that honey bees gather from different botanical sources and mix with nectar and bee secretions from its hypopharyngeal glands (such as B-glucosidase). Bee-pollen is recognized as an excellent dietary supplement and beneficial product in regard to health enhancing due to its content on phenolic compounds with antioxidant properties [3,6]. Furthermore, it is associated to natural source of dietary vegetable protein, becoming of a growing interest for the future as a substitute for meat, in order to consume healthier products and to reduce livestock farming as a reason for climate change.

Fresh bee-pollen has high microbial contamination which is potentially harmful to humans [7,8,9,10,11,12]. For this reason, it is often marketed as dried pollen. Hot-air drying process is an excellent tool to monitor its likely high health risk [13]. In return, dried pollen acquires a rough texture which often leads consumers to reject it. However, drying techniques adversely affect its quality characteristics and properties [3,14,15,16]. Canale et al. (2016) [17] proposed microwave drying under vacuum to reduce negative effects, but this technique has not yet been extensively studied.

In light of the above, by marketing bee-pollen as freshly frozen against dried, it helps to further preserve its properties and initial textures and flavors (reviewed by Campos et al., 2008) [2]. However, freezing does not reduce the initial potentially hazardous loads of microorganisms [13,18]. Furthermore, any failure in the cold-chain integrity, either temporary or permanent, can lead to microbial growth which poses a potentially significant safety threat to public health, in contrast, microbial load could be markedly reduced by applying a shock treatment before freezing. Ionizing radiation is an optional shock treatment for bee-pollen irradiation [8,12,19]. Although ionizing radiation is considered a safe technology, it is rather expensive, and consumers’ willingness and preference move to purchase organic food products instead. It is also difficult the logistic capacity to ship the fresh bee-pollen to the radiation processing plants, as often there are very few of them and are remotely located. For these reasons, it is important to implement new methods or technologies for treating frozen bee-pollen which guarantee safety for human consumption and that keep, as best as possible, sensory attributes and nutritional properties. A promising treatment for sterilization of beehive material is the double inductively coupled low pressure plasma that could be applied to the bee pollen decontamination [20].

In 2001, The U.S. Food and Drug Administration (FDA) formally approved the use of ozone as an antimicrobial agent in food [21]. It enabled to raise its use for the treatment, storage, processing, and packaging of foods together with the storage of raw foodstuffs for commercial purposes. The use of ozone became strengthened in comparison to other decontamination methods for foodstuffs because the only result of ozone when it decomposes is oxygen, it leaves no residues in the food or environment. Another advantage is that ozone is effective in removing bad odors and allergens from the air [22]. This makes ozone a possible candidate for microbial control in bee-pollen including mycotoxins [23,24]. However, there is little research devoted to its use on its product. Yook et al. (1998) [8] observed lower microbial load reduction in bee-pollen due to ozone exposure when compared to gamma-radiation, considering ozone unsatisfactory. These research were carried out under extremely specific and restricted conditions and hence, insufficient to discard ozone treatment in bee-pollen. Consequently, the main aim of this work is the evaluation of the methods for microbial decontamination of bee-pollen based on ozone exposure.

Not just reducing the microbial load of fresh or frozen bee-pollen is required for safe consumption, but also keeping it the lowest for the maximum period is essential. Microbial load is a limiting factor to set the shelf-life of this product. Cold chain maintenance is crucial to avoiding the microbial growth in non-dried bee-pollen. Although usually the fresh bee-pollen is kept in a frozen state, in this study monitoring of the microbial load exposed to ozone and keeping at refrigeration temperatures are carried out.

Moreover, ozone acts as a potential oxidizing agent. Yook et al. (1998) [8] noted certain effect of ozone on fatty acid composition such as a remarkable decrease of unsaturated fatty acid, and visual changes in pigments. Bearing in mind that polyphenols are biologically active bee-pollen components, and they are of a great biological interest because of their antioxidant activity [25], the effect of ozone treatment on them was also studied in our current work.

Finally, drying process of bee-pollen modifies its sensory characteristics making it more stringy and less palatable. These changes were another reason to carry out the present study. Together with the reduction of microbial load in this product to make it safe for human consumption, an evaluation of its sensory attributes was carried out comparing dried bee-pollen to fresh bee-pollen exposed to ozone and frozen afterwards.

## 2. Materials and Methods

### 2.1. Sampling of Bee Pollen

During spring 2016, samples of bee-pollen were gathered from an experimental apiary of the University of Córdoba (Córdoba, Spain) (37°55′33.5″ N, 4°43′26.1″ W). The bee colonies (*Apis mellifera iberiensis*) were housed in Langstroth hives placed on platforms raised 50 cm above ground level and were fitted with pollen-traps. Samples were collected every two days and kept frozen (−20 °C) up to their analysis.

### 2.2. Microbiological Quality

Ten grams of each bee-pollen sample were homogenized into 90 mL of peptone water solvent and decimal dilutions were prepared using the same solvent. For microbiological determination the methodology proposed by Estevinho et al. (2012) [26] was followed. Aerobic mesophilic bacteria were counted onto standard plate count agar (PCA) and incubated at 30 °C for 72 h. For molds and yeasts counts potato dextrose agar (PDA) was used and incubated at 25 °C for 5 days. *Staphylococcus aureus* was determined using Baird Parker agar and incubated at 37 °C for 48 h. Total *Enterobacteriaceae* was counted onto violet, red bile glucose agar (VRBG) incubated at 37 °C for 24 h. Finally, total coliforms were determined on brilliant green bile lactose incubated at 31 °C for 24 and 48 h. Determinations for all microbiological analysis were carried out in duplicate.

### 2.3. Bee-Pollen Treatments

In this study five different issues were raised: (i) Hot-air dried bee-pollen treatment, (ii) bee-pollen exposed to ozone treatment, (iii) evaluation of the shelf-life of bee-pollen exposed to ozone, (iv) study of changes on polyphenols present in bee-pollen exposed to ozone, (v) sensory evaluation of processed bee-pollen. For each of these trials, 10 g of sample were needed.

#### 2.3.1. Hot-Air Dried Bee-Pollen Treatment

For this trial a bee-pollen dryer was used (Mauro Valla dryer, Borgo Val di Taro, Italy). First, four groups were set up. Each group consisted of six samples of 10 g. Bee-pollen samples from three of the groups were dried at a temperature of 42 °C during 15, 30 and 45 min, respectively. The fourth group was kept as control and was not treated. Microbiological count was carried out before and after the drying process, as indicated above.

#### 2.3.2. Bee-Pollen Exposed to Ozone Treatment

For this assay an ozone generator, with ozone output of 200 mg/h, was used (Ozonoplus-10. Vida-10. China). The evaluations were performed on three groups consisting of 20 samples each (10 g/sample). Two groups were exposed to an ozone density of 200 mg/h for 30 and 60 min, respectively, within a polyethylene container. These timings of treatment were selected according to available literacy and previous non published assays. The third group was taken as control and was not exposed. The microbial counting was carried out before and after ozone exposition as described above.

### 2.4. Evaluation of the Shelf-Life of Bee-Pollen Exposed to Ozone

In this trial 18 samples of 10 g of bee-pollen were treated with ozone for 30 min as described above and kept in a refrigerator at 4 °C. These samples were put under microbiological analyses consisting of six evaluations: Fresh bee-pollen, just treated bee-pollen, and 1, 2, 3, and 6 weeks after exposition. Microbial counting was executed as described above.

### 2.5. Evaluation of Polyphenols Present in Bee-Pollen

Ten samples of bee-pollen were used in this trial. From each sample three subsamples were obtained. First subsamples were dried for 30 min as described above. The second one was exposed to ozone for 30 min and the third group was set as control, was not exposed. All samples were frozen afterwards up to their analyses. In every group the presence and concentration of 11 polyphenols belonging to different structural groups was evaluated; three phenolic acids: gallic acid (hydroxybenzoic acid), caffeic acid, and p-coumaric acid (hydroxycinnamic acids) and seven aglycon flavonoids: two flovones (Luteolin and Chrysin), three flavanones (Quercetin, Rutin (quercetin 0–3-rutoside) and kaempherol), one flavonol (Naringerin), one isoflavones (Genistein), and one trihydroxy-stilbene (resveratrol) using Liquid Chromatography-Mass Spectrometry(LC-MS/MS). The pollen samples were homogenized by grinding to a fine powder, using a common mortar. Next, 1 g of pollen was mixed with 10 mL of ethanolic solution (Ethanol: water in proportion of 80:20 *v*/*v*) in a polypropylene tube and sonicated in an ultrasonic water bath for 20 min at room temperature. Afterwards, centrifugation was carried out at 4000 rpm during 10 min and the supernatant was transferred to a 50 mL flask. This procedure was repeated two times more and the supernatants were combined and brought to 50 mL final volume with ethanol/water (80/20 *v*/*v*). To 0.8 mL of extract filtrated through a 22 µm nylon filter, 0.2 mL of a water solution with 0.1% of acid formic was added before quantitation in High Performance Liquid Chromatography-Mass Spectrometry (HPLC-MS/MS), whose working conditions are described in Supplementary information.

The identity of compounds was assessed by comparing their ions transition in MS/MS, retention time, and ratio SRM1/SMR2 (Selected Reaction Monitoring) with that of standards. Quantitation was carried out by a standard addition method in the range of 20 to 800 ng/mL with good linearity (r^2^ > 0.995) for each compound in each pollen sample. Recoveries ranged from 71.2 to 111.6% for all polyphenols at 30 and 500 ng/mL fortification levels and the quantification limits (LOQs) calculated on the basis of ten times the signal-to-noise ratios were lower than 0.8 µg/g.

### 2.6. Sensory Evaluation of Processed Bee-Pollen

Sensory analysis was conducted on untreated fresh bee-pollen; 30 min ozone exposed bee-pollen; 15, 30, and 45 min hot-air dried bee-pollen, following Serra Bonvehí and Gómez Pajuelo (1988) [27] and Baldi et al. (2004) [28] analysis. Visual, texture, olfactive and taste attributes were evaluated.

Panel of trained and experienced assessors described color, odor intensity and its descriptors, basic flavors, aftertaste, persistence, aroma intensity and its descriptors, tactile and mouth textures.

### 2.7. Data Analysis

Data were statistically processed using SPSS (Statistical Package for the Social Sciences) Statistics software for Windows, IBM Corp, 2016. Version 24.0. IBM Corp, Armonk, NY, USA. All the variables available were tested to check whether data violated the assumptions for regular parametric tests to report valid results. Parametric statistics were applied when possible. When data resulted non normally distributed, or there was no variance homogeneity (heteroscedasticity), non-parametric statistics were used. The tests are specified in the results.

## 3. Results

None of the variables of the study of trials 1, 2, and 3 were normally distributed (Shapiro–Wilk, *p* < 0.05) (Table 1). For this reason, non-parametric statistical analysis was applied.

### 3.1. Trial 1. Heat Treatment: Hot-Air Dried Bee-Pollen

In every treatment a significant microbial reduction was observed in comparison to the untreated control group (Wilcoxon test, *p* < 0.05). However, no significant differences were found within the three exposures timing groups. The greatest reduction was noticed on *Enterobacteriaceae*, showing percentages above 90%, followed by molds and yeasts 70–80% reduction and finally mesophilic aerobes 53–67% reduction. All results, as well as reduction percentage are shown in Table 2.

### 3.2. Trial 2. Bee-Pollen Exposed to Ozone Treatment

All treatments showed a significant microbial reduction in comparison to the untreated control group. Otherwise, there was no significant difference between the two different timing exposures (Wilcoxon test, *p* > 0.05). For *Enterobacteriaceae* the contamination reduction observed was between 80.0% and 90.9%, for mesophilic aerobes was 69.1% and 83.1%, and for molds and yeasts between 89.4% and 89.5%. All results, as well as reduction percentage are shown in Table 3.

### 3.3. Evaluation of the Shelf-Life of Bee-Pollen Treated Combining Hot-Air Drying (15 min) and Ozone Exposure (30 min)

No significant differences were found between ozone exposure treatment timing 30 and 60 min to reduce microbial load. Thus, the lowest timing exposure to ozone together with hot-air drying during 15 min was chosen to evaluate the shelf-life of treated bee-pollen and cold storage (4 °C).

Using this treatment, evolution of microbial load was studied in 18 samples in order to obtain helpful information, which could be used in the future, to set shelf-life of bee-pollen treated according to this method. After this treatment, results showed an important and remarkable reduction in all microbiological groups included in this study, especially in *Enterobacteriaceae* group. Three weeks after bee-pollen has been treated and kept cool, microbial load increased slowly. Last evaluation was carried out six weeks after treatment and although the microbial load continued increasing, it was held at a lower level than initially observed in fresh bee-pollen. Results are shown in Table 4.

### 3.4. Study of Changes on Polyphenols Present in Bee-Pollen after Treatment (Heat and Ozone) during 30 min

Potential changes in polyphenols composition were evaluated. Comparison between fresh bee-pollen, hot-air dried bee-pollen (30 min) and ozone exposed bee-pollen (30 min). To analyze the results, non-parametric statistics was applied as data resulted non normally distributed, there was no variance homogeneity (heteroscedasticity), and number of samples was scarce. Results were not normally distributed (Shapiro–Wilk, *p* < 0.05). No significant differences were detected within the three types of bee-pollen: fresh, hot-air dried, or ozonized for polyphenols studied (Friedman test, *p* < 0.05). Results are shown in Table 5. It is necessary to point out, that flavonoids are present in pollen in greater amount that phenolic acid, in accordance with our results, and are mainly in the form of glycosides, molecules bond to a sugar group. In this work, hydrolysis was not employed in the sample preparation procedure to quantified aglycons but the natural free aglycons in each pollen were determined. The hypopharyngeal gland secretions of the honeybee, cause partial enzymatic hydrolysis of glycosides to free aglycons during pollen collection. The presence of free aglycones is a good indicator of the quality of pollen because the glycoside bond reduces antioxidative properties [29].

### 3.5. Sensory Evaluation of Processed Bee-Pollen

For all evaluated parameters, bee-pollen exposed to ozone during 30 and 60 min showed no sensory differences in comparison to untreated fresh bee-pollen. However, the results obtained revealed sensory differences between fresh bee-pollen and hot-air dried bee-pollen (during 15, 30, and 45 min), except for the color attribute which showed no appreciable change. In Table 6 sensory profile for each treatment is presented.

## 4. Discussion

Microbiological contamination of commercial bee-pollen arises from different sources. One of them is the plant from which it comes from. Later, the honey bee handles the pellets of pollen to set them on their legs. Next hazardous step is the collection of bee-pollen using pollen-traps and eventually, final handling of bee-pollen to market it [9,30]. When the bee-pollen reaches the market, its microbiological quality must be guaranteed to be considered a safe product intended for human consumption, but also its nutritional properties must be preserved, as well as its value as a dietary supplement and palatability for consumers.

Regarding food safety, our work studied the three more frequent microbiological groups considered in this kind of research [2,31]. Taking note with special interest to *Enterobacteriaceae*, some agents responsible for health issues in human beings are included within this group. *Enterobacteriaceae* load in bee-pollen has a double origin. On the one hand, they can contaminate the bee-pollen through the environment, the activity of the honey bee that moves all over the surroundings, for example when they visit a source of polluted water used by other animals. On the other hand, *Enterobacteriaceae* are the main part of the microbiota presented in the intestine of healthy honey bees and they are also used as a hygiene indicator of the handling procedure of the product.

By contrast, the presence of mesophilic aerobes has a remarkable relation with initial contamination of raw materials, as well as the tools and materials used all along the production, handling, and storage of bee-pollen. Finally, molds and yeasts load is linked to environmental conditions [31,32].

Initially, untreated bee-pollen samples present in every trial a high load of *Enterobacteriaceae* potentially dangerous for consumption. Moreover, this microbiological group presented the best response when treating the product.

Usual hot-air drying treatment is efficient to reduce the microbial load [13], as it was observed in our trial, in which the reduction of *Enterobacteriaceae* was significant and the percentage of reduction for mesophilic aerobes, molds, and yeasts also was medium or even high (see Table 2). For this reason, hot-air drying should be satisfactory to achieve this purpose, but it also entails some inconveniences such as the loss of nutritional properties, reduction of palatability and consumers’ rating [14,15]. In this sense our objective was to study different efficient methods that let avoiding the inconveniences of hot-air drying.

Not only freezing the fresh bee-pollen is sufficient to guarantee its food safety, while it stops the growth of microorganisms, it does not reduce the present ones. For this reason, it would be a suitable option to adopt a shock treatment followed by cold storage. In the present work, the studied treatment was ozone exposure. Our results show an important reduction of microbial load when bee-pollen is exposed to ozone (see Table 3) so it could be considered a convenient shock treatment. Posterior freezing preservation (−20 °C) would avoid the subsequent increase of bacterial load, making it a safer product. Furthermore, ozone treatment allowed maintaining a high microbial load reduction when bee-pollen was kept in the refrigerator (4 °C) for several weeks (see Table 4), which offers new trade opportunities although further research is needed to ensure food safety.

In this work the effect of ozone on bee-pollen components was also raised, due to its oxidative capacity reported by other studies [8]. Preservation of nutritional qualities is crucial for this kind of product. Bee-pollen components, more frequently valued by consumers because of their antioxidant properties, such as polyphenols, were used as model [3,25,33]. Bee-pollen was treated with ozone for 30 min due to the significant reduction of microbial load in comparison to untreated control group of fresh bee-pollen. This ozone treatment was also chosen because no differences between 30 and 60 min ozone exposure were found. The obtained results indicate that ozone treatment did not have a negative impact on polyphenols evaluated in the present work (see Table 5) which makes ozone exposure a potential treatment for bee-pollen.

Finally, improving consumers acceptance of bee-pollen is another objective of this study. Sensory analysis carried out showed no differences between fresh and ozone exposed bee-pollen. However, hot-air dried bee-pollen presented a different sensory profile, except for the color, and the more the exposure to hot air, the more its sensory profile changes. Thus, the vegetation/green odor intensity for 15 min hot-air dried bee-pollen is similar to that of the fresh bee-pollen and most of the textural attributes are kept. However, the roasted flavor arises after the hot-air drying treatment and other flavor descriptors (humidity and floral) for fresh bee-pollen are lost. The persistence is shorter after the treatment.

Despite these promising results and the low cost of ozonation equipment, new research is required on the possible appearance of harmful compounds from oxidation caused by ozone, although we found authors who already point to these limitations [34].

## 5. Conclusions

The ozonation treatment achieved a contamination reduction of 80.0%–90.9%, 69.1%–83.1%, and 89.4%–89.5% for *Enterobacteriaceae,* mesophilic aerobes, and molds and yeasts, respectively. Since no significant differences were found between ozone exposure treatment timing 30 and 60 min to reduce microbial load, the lowest timing exposure to ozone together with hot-air drying during 15 min was chosen to evaluate the shelf-life of treated bee-pollen and cold storage (4 °C). Last evaluation was carried out six weeks after treatment and although the microbial load continued to increasing, it was held at a lower level than initially observed in fresh bee-pollen.

The obtained results indicate that ozone treatment did not have a negative impact on polyphenols evaluated in the present work which makes ozone exposure a potential treatment for bee-pollen. Sensory analysis carried out showed no differences between fresh and ozone exposed bee-pollen. However, hot-air dried bee-pollen presented a different sensory profile, except for the color, and the more the exposure to hot air, the more its sensory profile changes. Thus, the vegetation/green odor intensity for 15 min hot-air dried bee-pollen is similar to that of the fresh bee-pollen and most of the textural attributes are kept. However, the roasted flavor arises after the hot-air drying treatment and other flavor descriptors (humidity and floral) for fresh bee-pollen are lost. Moreover, the persistence is shorter after the treatment.

## Figures and Tables

**Table 1:** Shapiro–Wilk test for the study and distribution of the data, *p* < 0.05.

	Hot-Air Drying			Ozone			Combination		
	Statistic	df	Sig	Statistic	df	Sig	Statistic	df	Sig
*Enterobacteriaceae*	0.473	40	0.000	0.164	80	0.000	0.290	30	0.000
*Enterobacteriaceae* reduction percentage	0.616	40	0.000	0.691	80	0.000	0.642	30	0.000
Mesophillic aerobes	0.549	40	0.000	0.153	80	0.000	0.362	30	0.000
Mesophillic aerobes reduction percentage	0.873	40	0.000	0.761	80	0.000	0.898	30	0.008
Moulds & yeasts reduction percentage	0.785	40	0.000	0.688	80	0.000	0.831	30	0.000

df: degree of freedom; Sig: Significance.

**Table 2:** Microbial load reduction of hot-air dried pollen (42 ± 1 °C) exposed to three different timing exposure. The results are expressed as number of samples (N), average of CFU counted, and percentage of microbial load reduction (mean ± S.D.).

Microbiological Group	Hot-Air Dried Timing	N	Counting (log CFU/g) (Mean ± S.D.)	% Reduction of Contamination (Mean ± S.D.) *
*Enterobacteriace*	Without treatment	10	5.39 ± 5.34	0.0 ± 0.0 ^a^
	15 min	10	3.83 ± 3.66	93.8 ± 7.4 ^b^
	30 min	10	3.93 ± 4.07	96.1 ± 3.8 ^b^
	45 min	10	3.77 ± 4.02	96.9 ± 5.0 ^b^
Mesophillic aerobes	Without treatment	10	4.83 ± 4.82	0.0 ± 0.0 ^a^
	15 min	10	4.31 ± 4.33	53.0 ± 55.7 ^b^
	30 min	10	4.09 ± 3.91	67.2 ± 35.3 ^b^
	45 min	10	4.16 ± 3.86	59.1 ± 33.2 ^b^
Moulds & yeasts	Without treatment	10	7.54 ± 7.75	0.0 ± 0.0 ^a^
	15 min	10	5.89 ± 5.87	78.0 ± 32.5 ^b^
	30 min	10	5.97 ± 5.94	70.3 ± 45.8 ^b^
	45 min	10	5.89 ± 5.83	80. 8 ± 27.2 ^b^

* a, b Superscripts indicate significantly different results of the reduction percentage within the microbiological group (non-parametric statistic. Wilcoxon test, *p* < 0.05). S.D. means Standard Deviation.

**Table 3:** Microbial load reduction obtained from bee-pollen directly exposed to ozone (output: 200 mg/h). The results are expressed as number of samples (N), average of CFU counted, and percentage of microbial load reduction (mean ± S.D.).

Microbiological Group	Ozone Exposure Timing	N	Counting (log CFU/g) (Mean ± S.D.)	% Reduction of Contamination (Mean ± S.D.) *
*Enterobacteriaceae*	Without treatment	20	6.91 ± 7.36	0.0 ± 0.0 ^a^
	30 min	20	4.94 ± 5.02	80.0 ± 6.6 ^b^
	60 min	20	4.61 ± 4.67	90.9 ± 3.0 ^b^
Mesophillic aerobes	Without treatment	20	6.31 ± 6.80	0.0 ± 0.0 ^a^
	30 min	20	4.97 ± 5.16	69.1 ± 8.1 ^b^
	60 min	20	4.73 ± 4.95	83.1 ± 5.0 ^b^
Moulds & yeasts	Without treatment	20	8.88 ± 9.30	0.0 ± 0.0 ^a^
	30 min	20	6.32 ± 6.31	89.5 ± 4.4 ^b^
	60 min	20	6.29 ± 6.54	89.4 ± 4.2 ^b^

* a, b Superscripts indicate significantly different results of the reduction percentage within the microbiological group (non-parametric statistic. Wilcoxon test, *p* < 0.05).

**Table 4:** Evaluation of the microbial load, at six different times, in bee-pollen treated mixing 30 min ozone exposure together with 15 min hot-air drying and kept in a refrigerator at 4 °C. The results are expressed as number of samples (N) and average of CFU counted (mean ± S.D.).

	*Enterobacteriaceae*		Mesophillic Aerobes		Moulds & Yeasts	
	N	(mean ± S.D.) *	N	(mean ± S.D.) *	N	(mean ± S.D.) *
b.t. ^1^	18	6.78 ± 7.15 ^a^	18	6.02 ± 6.54 ^a^	18	8.39 ± 8.82 ^a^
Just a.t. ^2^	18	4.03 ± 4.54 ^b^	18	4.16 ± 4.44 ^b^	18	5.81 ± 6.06 ^b^
1 week a.t. ^2^	18	3.99 ± 4.30 ^c^	18	4.56 ± 4.83 ^b,c^	18	6.63 ± 7.07 ^b,c^
2 weeks a.t. ^2^	18	4.42 ± 4.64 ^d^	18	4.39 ± 4.70 ^b^	18	6.90 ± 7.29 ^c,d^
3 weeks a.t. ^2^	18	4.55 ± 4.80 ^e,f^	18	4.77 ± 5.11 ^b^	18	6.92 ± 7.28 ^c,d^
6 weeks a.t. ^2^	17	5.37 ± 5.77 ^e,f^	17	4.98 ± 5.15 ^c^	17	7.05 ± 7.33 ^d^

* a,b,c,d,e,f, Superscripts indicate significantly different results of the reduction percentage within the microbiological group (non-parametric statistic. Wilcoxon test, *p* < 0.05). ^1^ b.t.: before treatment. ^2^ a.t.: after treatment.

**Table 5:** Polyphenols in bee-pollen. The presence of polyphenols was quantified in bee-pollen samples: 10 initial samples of fresh-frozen bee-pollen, 10 hot-air dried bee-pollen samples, and 10 ozone exposed bee-pollen samples. The results are presented as number of samples that contain the specific polyphenols (N) and the average (Mean ± S.D.) (µg/g dry) for each of polyphenols studied. Gallic Acid, Genistein, and Resveratrol were found below the Limit of Quotation (LOQ) in all the analyzed samples.

Polyphenols	Caffeic		Chrysin		Kaempher		Luteolin		Naringerin		P-Coumaric		Quercetin		Rutin	
	N	Mean ± S.D.	N	Mean ± S.D.	N	Mean ± S.D.	N	Mean ± S.D.	N	Mean ± S.D.	N	Mean ± S.D.	N	Mean ± S.D.	N	Mean ± S.D.
Fresh Pollen	5	3.3 ± 2.3	3	4.5 ± 4.5	5	1.9 ± 0.5	5	63.1 ± 41.9	4	2.1 ± 1.1	5	7.1 ± 4.8	5	7.5 ± 1.9	5	6.7 ± 2.7
Dried Pollen	5	3.7 ± 1.8	3	4.4 ± 3.7	5	1.6 ± 0.8	5	70.6 ± 47.7	4	2.7 ± 0.9	5	7.1 ± 4.4	5	7.7 ± 1.4	5	6.3 ± 2.9
Ozonize Pollen	5	3.5 ± 2.1	3	6.0 ± 4.7	5	1.7 ± 0.4	5	86.3 ± 70.7	4	2.6 ± 1.9	5	7.8 ± 5.4	5	7.9 ± 2.6	5	6.6 ± 2.7

**Table 6:** Sensory profiles of fresh and hot-air dried pollen.

Bee-Pollen Type	Sensory Profile
Fresh (untreated)	Intense (5) odour (vegetation/green); grains change their shape when pressing with the fingers and combine together in one mass, pollen dust is observed, very wet in mouth, sweet, bitter, slightly salted, and lightly acid. Medium (3) flavor (vegetation/green/humidity, floral) and medium (3) persistence.
15′ Dried pollen	Intense (5) odor (vegetation/green); grains change their shape when pressing with the fingers but not combine together in one mass, no pollen dust is observed; wet in mouth, sweet, and slightly salted. Medium (3) flavor (vegetal/green/roasted) and short (2) persistence.
30′ Dried pollen	Medium (3) odor (vegetation/green); grains change their shape when pressing with the fingers but not combine together in one mass, no pollen dust is observed; wet in mouth, sweet and slightly salted. Soft (2) flavor (vegetation/green/roasted) and short (2) persistence.
45′ Dried pollen	Medium (3) odor (vegetation/green); grains change their shape when pressing with the fingers, no pollen dust is observed; dry in mouth, slightly salted. Very soft (1) flavor (vegetation/green/roasted) and short (2) persistence.

## Supplementary Materials

The following are available online at https://www.mdpi.com/article/10.3390/foods10112593/s1, Table S1: Gradient of mobile phase. Table S2: Characterization of the phenolic compounds by LC-MS/MS. Table S3: Recoveries (%), precision (RSD%) and quantitation limit (LOQ).

## References

[1] Jean-Prost P., Médori P., Le Conte Y. (2005). Apiculture: Connaître L’abeille, Conduire le Rucher.7 Édition Revue et Complétée.

[2] Campos M.G.R., Bogdanov S., de Almeida-Muradian L.B., Szczesna T., Mancebo Y., Frigerio C., Ferreira F. (2008). Pollen composition and standardisation of analytical methods. J. Apic. Res..

[3] Campos M.G.R., Frigerio C., Lopes J., Bogdanov S. (2010). What is the future of Bee-Pollen?. J. ApiProduct ApiMedical Sci..

[4] Brodschneider R., Crailsheim K. (2010). Nutrition and health in honey bees. Apidologie.

[5] Wright G.A., Nicolson S.W., Shafir S. (2018). Nutritional Physiology and Ecology of Honey Bees. Annu. Rev. Entomol..

[6] Muniategui S., Sancho T., Terradillos L., Huidobro J., Simal-Lozano J. (1993). Composición del polen apícola. Vida Apícola.

[7] Bonvehí J.S., Jordà R.E. (1997). Nutrient Composition and Microbiological Quality of Honeybee-Collected Pollen in Spain. J. Agric. Food Chem..

[8] Yook H.-S., Lim S.-I., Byun M.-W. (1998). Changes in Microbiological and Physicochemical Properties of Bee Pollen by Application of Gamma Irradiation and Ozone Treatment. J. Food Prot..

[9] Hani B., Dalila B., Saliha D., Daoud H., Mouloud G., Seddik K. (2012). Microbiological Sanitary Aspects of Pollen. Adv. Environ. Biol..

[10] Puig-Peña Y., del-Risco-Ríos C.A., Álvarez-Rivera V., Leiva-Castillo V., García-Neninger R. (2012). Comparación de la calidad microbiológica del polen apícola fresco y después de un proceso de secado. CENIC Cienc. Biológicas.

[11] Nardoni S., D’Ascenzi C., Rocchigiani G., Moretti V., Mancianti F. (2015). Occurrence of moulds from bee pollen in Central Italy—A preliminary study. Ann. Agric. Environ. Med..

[12] Hosny A.S., Sabbah F.M., EL-Bazza Z.E. (2018). Studies on the microbial decontamination of Egyptian beepollen by γ radiation. Egypt. Pharm. J..

[13] Mauriello G., De Prisco A., Di Prisco G., La Storia A., Caprio E. (2017). Microbial characterization of bee pollen from the Vesuvius area collected by using three different traps. PLoS ONE.

[14] Collin S., Vanhavre T., Bodart E., Bouseta A. (1995). Heat Treatment of Pollens: Impact on Their Volatile Flavor Constituents. J. Agric. Food Chem..

[15] Bonvehí J.S., Torrentó M.S., Lorente E.C. (2001). Evaluation of Polyphenolic and Flavonoid Compounds in Honeybee-Collected Pollen Produced in Spain. J. Agric. Food Chem..

[16] Domínguez-Valhondo D., Gil D.B., Hernández M.T., González-Gómez D. (2011). Influence of the commercial processing and floral origin on bioactive and nutritional properties of honeybee-collected pollen. Int. J. Food Sci. Technol..

[17] Canale A., Benelli G., Castagna A., Sgherri C., Poli P., Serra A., Mele M., Ranieri A., Signorini F., Bientinesi M. (2016). Microwave-Assisted Drying for the Conservation of Honeybee Pollen. Materials.

[18] Kaèániová M., Fikselová M., Hašèík P., Kòazovická V., Nôzková J., Fatrcová-Šrámková K. (2010). Changes in microflora of bee pollen treated with uv light and freezing during storage. Ecol. Chem. Eng. A.

[19] Kędzia B., Hołderna-Kędzia E. (2010). The microbiological decontamination of pollen by using of ionizing radiation. Postępy Fitoter..

[20] Priehn M., Denis B., Aumeier P., Kirchner W.H., Awakowicz P., Leichert L.I. (2016). Sterilization of beehive material with a double inductively coupled low pressure plasma. J. Phys. D Appl. Phys..

[21] Rice R.G., Graham D.M. (2001). US FDA regulatory approval of ozone as an antimicrobial agent–what is allowed and what needs to be understood. Ozone News.

[22] Rice R.G., Graham D.M., Lowe M.T. (2002). Recent Ozone Applications in Food Processing and Sanitation. Food Saf. Mag..

[23] Beuchat L.R., Chmielewski R., Keswani J., Law S.E., Frank J.F. (1999). Inactivation of aflatoxigenic Aspergilli by treatment with ozone. Lett. Appl. Microbiol..

[24] Zhu F. (2018). Effect of ozone treatment on the quality of grain products. Food Chem..

[25] Kroyer G., Hegedus N. (2001). Evaluation of bioactive properties of pollen extracts as functional dietary food supplement. Innov. Food Sci. Emerg. Technol..

[26] Estevinho L.M., Rodrigues S., Pereira A.P., Feás X. (2011). Portuguese bee pollen: Palynological study, nutritional and microbiological evaluation. Int. J. Food Sci. Technol..

[27] Serra Bonvehí J., Gómez Pajuelo A. (1988). La calificación de mieles mediante el análisis organoléptico. Apiacta.

[28] Baldi Coronel B., Grasso D., Chaves Pereira S., Fernández G. (2004). Caracterización bromatológica del polen apícola argentino. Cienc. Docencia Tecnol..

[29] Cook N.C., Samman S. (1996). Flavonoids—Chemistry, metabolism, cardioprotective effects and dietary sources. J. Nutr. Biochem..

[30] González G., Hinojo M.J., Mateo R., Medina A., Jiménez M. (2005). Occurrence of mycotoxin producing fungi in bee pollen. Int. J. Food Microbiol..

[31] De Arruda V.A.S., Dos Santos A.V., Sampaio D.F., Araújo E.D.S., Peixoto A.L.D.C., Estevinho M.L.M.F., de Almeida-Muradian L.B. (2017). Microbiological quality and physicochemical characterization of Brazilian bee pollen. J. Apic. Res..

[32] Gliński Z., Jarosz J. (1988). Mikroflora pszczoły miodnej. Post Mikrobiol..

[33] Almaraz-Abarca N., Campos M.G., Ávila-Reyes J.A., Naranjo Jiménez N., Herrera-Corral J., González-Valdez L.S. (2004). Vari-ability of antioxidant activity among honey bee-collected pollen of different botanical origin. J. Sci. Technol. Am..

[34] Kim J.-G., Yousef A.E., Dave S. (1999). Application of Ozone for Enhancing the Microbiological Safety and Quality of Foods: A Review. J. Food Prot..

