# Supplementary material for: Microbial Decontamination of Bee Pollen by Direct Ozone Exposure
Source: Foods. 2021 Oct 27;10(11):2593. doi: 10.3390/foods10112593 (PMC8623575; doi:10.3390/foods10112593)
Supplement: Supplementary file 1 [file foods-10-02593-s001.zip › foods-1420497-supplementary.pdf]

Quantification

An Agilent system with a Model 1200 chromatograph and a Model 6410 triple quadrupole analyser (Agilent Technologies, Palo Alto, CA, USA) were employed. LC analysis was performed with a Kinetex 2.6µm biphenyl column of 100 x 3 mm i.d. and, 100Å particle size (Phenomenex, Torrance, CA, USA). The mobile phase A was 0.1% formic acid in water and the mobile phase B was MeOH following the gradient indicated in Table S1. The column was maintained at 25ºC, flow rate varied between 0.2 and 0.35 mL min^{-1} and the injection volume was 10 µL. The system used an electrospray ion source (ESI) operating in negative mode in the following conditions: drying gas temperature (325ºC), drying gas flow (9 $Lmin^{-1}$), pressure of the nebulizer (35 psi) and capillary voltage (-4000 V). Nitrogen gas was used in the nebulizer and in the collision cell. Identification of each polyphenol was based on the detection of two selected reaction monitoring (SRMs transitions) one quantifier transition (SRM1) and one qualifier transition (SRM2) by flow injection analysis of each standard analyte (Table S2).

Table S1. Gradient of mobile phase.

Time(min)	MeOH	0.1% Formic acid	Flow(mL.min^{-1})
0	5	95	0.35
6	50	50	0.35
9.5	55	45	0.35
10.5	55	45	0.20
12.0	57	43	0.20
15.0	67.5	32.5	0.20
17.0	75.0	25	0.3
22	90	10	0.3
25	5	95	0.35
35	5	95	0.35

Table S2. Characterization of the phenolic compounds by LC-MS/MS.

Time segment	Compound	Precursor	SRM1 SMR2	Fragmentor (V)	Collision Energy (V)	Dwell
1	Gallic acid	169	125<br>79	80<br>80	10<br>20	500<br>400
2	Rutin	609	301<br>271	180<br>180	10<br>25	120<br>120
2	Caffeic acid	179	135<br>89	80<br>80	10<br>25	140<br>140
2	p-Coumaric acid	163	119<br>93	80<br>80	15<br>30	120<br>120
3	Quercetin	301	151<br>179	140<br>140	13<br>10	55<br>50

3	Kaempherol	285	117<br>211	160<br>160	55<br>30	55<br>50
3	Luteolin	285	133<br>175	100<br>100	30<br>17	55<br>50
3	Naringenin	271	119<br>107	100<br>100	30<br>25	50<br>55
3	Genistein	269	133<br>63	100<br>100	33<br>40	55<br>50
3	Chrysin	253	143<br>107	90<br>90	30<br>25	55<br>50
3	Resveratrol	227	143<br>185	110<br>110	25<br>13	55<br>50

Table S3. Recoveries (%), precision (RSD%) and quantitation limit (LOQ) .Recoveries are the mean of three independents determination at 30 and 500 ng/ml, equivalent at 1.8 and 31.2 µg/g.

	Recovery (average (R) and RSD				LOQ (µg/g)
	30ng/ml		500ng/ml		
	% R	% RSD	% R	% RSD	
Caffeic	71.2	12	75.8	10	0.14
Galic	89.5	9	100.7	6	0.6
p-Coumaric	80.7	8	89.8	1	0.17
Chrysin	83.05	4	89.5	4	0.36
Kaempherol	96.5	20	80.7	16	0.37
Luteolin	103.3	5	98.75	1	0.66
Naringerin	97.3	8	84.2	5	0.71
Quercetin	101.9	11	90.85	6	0.67
Rutin	111.6	10	103	1	0.38
Resveratrol	74.25	15	90.05	14	0.5
Genistein	88.1	16	100.4	13	0.2
